# A Review of the Prostatic Urethral Lift for Lower Urinary Tract Symptoms: Symptom Relief, Flow Improvement, and Preservation of Sexual Function in Men With Benign Prostatic Hyperplasia

**DOI:** 10.1007/s11884-015-0296-5

**Published:** 2015-03-27

**Authors:** Neal Shore

**Affiliations:** Carolina Urologic Research Center, 823 82nd Parkway, Suite B, Myrtle Beach, SC 29572 USA

**Keywords:** Benign prostatic hyperplasia, Lower urinary tract symptoms, Minimally invasive therapy, Sexual function, Quality of life, Surgery

## Abstract

Prostatic urethral lift (PUL) has been shown to be a safe, effective treatment option for lower urinary tract symptoms secondary to benign prostatic hyperplasia. Discomfort with PUL is similar to that with rigid cystoscopy and can be tolerated with local anesthesia in an office setting. Of those who are given voiding trials, 70–80 % of subjects do not require a catheter. Subjects often quickly return to pre-operative activity level with minimal absence from work. Symptom relief can start within 2 weeks and be sustained through 2 years. Urinary flow rate improvements have been shown to be durable through 2 years. The most common adverse effects are dysuria, hematuria, pain, and urgency which are typically mild to moderate and transient. Sexual function appears to be preserved after PUL with no reported new-onset erectile dysfunction or anejaculation events. The retreatment rate is reported to be 7.5 % at 2 years.

## Introduction

Lower urinary tract symptoms (LUTS) associated with benign prostatic hyperplasia (BPH) rank number 4 (13.5 %) among the top 10 diagnosed diseases in men aged 50 years and older, surpassed only by coronary artery disease/hyperlipidemia, hypertension, and type 2 diabetes mellitus [1]. Chronic LUTS often lead to loss of sleep, social isolation, and depression, thus having a significant impact on a patient’s quality of life [2]. The majority of patients opt for treatment with medications. Approximately 30 % of men initiating BPH medication however discontinue treatment early due to insufficient relief and bothersome side effects [3]. There remains a need for a BPH treatment that can address LUTS better than medications yet is associated with less morbidity than current procedures.

Medical therapy with alpha-blockers, 5-alpha-reductase inhibitors, or tadalafil, a phosphodiesterase type 5 inhibitor, offers a modest 3- to 6-point improvement in International Prostate Symptom Score (IPSS) and can be associated with bothersome side effects, such as sexual dysfunction, dizziness, and chronic fatigue (asthenia) [4]. Combination therapy can be more effective in enlarged prostates, but side effects including erectile dysfunction and decreased libido are additive. As with many treatment paradigms, the most potent treatments for LUTS are also associated with the highest risks. Transurethral resection of the prostate (TURP), first introduced in the 1930s, is considered the gold standard surgical therapy for BPH [5]. Although highly effective in relieving LUTS, it has a 20 % rate of perioperative morbidity and long-term complications that include ejaculatory dysfunction (65 %), erectile dysfunction (10 %), urinary incontinence (3 %), need for transfusion (8 %), TUR syndrome (3 %), serious cardiovascular events (2 %), as well as urethral stricture and bladder neck contracture (7 %) [4, 6, 7]. New laser-based modalities such as photoselective vaporization (PVP) may offer less bleeding and eliminate TUR syndrome but are otherwise associated with morbidity rates similar to TURP [8–10]. IPSS improvement for TURP or laser procedures ranges 12 to 15 points [4, 9].

Less invasive thermal therapies, namely transurethral microwave therapy (TUMT) and transurethral needle ablation (TUNA), offer the ability to treat patients using local or regional anesthesia and reduce the likelihood of the serious complications seen with tissue removal procedures. Thermal therapies offer a mean IPSS improvement of 9 to 12 points but have been associated with urinary retention, routine catheterization, ejaculatory dysfunction, and prolonged irritative symptoms after the procedure [4, 6, 9].

The prostatic urethral lift (PUL) procedure has been introduced with the goal to better address the needs of a minimally invasive BPH procedure, namely one that is able to be performed routinely under local anesthesia, with a lower rate of post-operative catheter usage, more rapid symptom relief with less morbidity, and preservation of sexual function. Approved in Europe in 2009, Australia in 2010, and now the USA in 2013, PUL has been studied in several trials. This review is intended to synthesize the data to examine the risks and benefits of this new therapy that has entered the urologist’s BPH treatment armamentarium.

## Evidence Analysis

All studies with an estimate of absolute change and either a 95 % confidence interval for the change or a standard deviation were included in the analysis. The estimates were combined across studies using the inverse of the variances for individual studies for weighting [11]. Per the Fleiss method, homogeneity was assessed, and where the results were found to be significantly heterogeneous, the combined estimate of change was calculated using the heterogeneity adjustment. *P* values were calculated to assess the statistical significance of the combined estimate of absolute change from baseline using the calculated combined estimate of change and standard errors, using the normal distribution.

## PUL Surgical Technique

The PUL procedure involves transurethrally placing permanent UroLift® implants (NeoTract, Inc., Pleasanton, CA) under cystoscopic guidance into the prostate gland. The urologist inserts the handheld delivery device through a sheath and compresses the prostate lobe with the delivery instrument to achieve a “de-obstructed” effect. Then, the implants are delivered through a 19-gauge needle that traverses the prostate gland from the urethra to the capsule. At the end of a suture housed in the needle, a small metallic tab captures the prostate capsule and the suture is tensioned. A urethral end piece is then affixed to the suture, which is then severed. In such a way, each implant is individually sized in situ to the width of the compressed lobe at that location. Because the glandular tissue is more compliant than the fibro-muscular capsule, the implant lifts the urethra toward the capsule, retracting the obstructing lateral lobe and enlarging the urethral lumen.

## Safety, Effectiveness, and Durability

The PUL procedure was first shown to be safe and feasible by Woo et al. in a prospective, nonrandomized study of 19 patients at two Australian centers that started in 2005 [12]. This study was expanded to more centers and included 64 subjects who were followed to 2 years [13]. Subjects were at least 55 years old with moderate to severe symptomatic LUTS (Table 1). Exclusion criteria included obstructive median lobe, active urinary retention, urinary tract infection, urinary calculus, or suspicion of prostate cancer. Subjects were evaluated at 0.5, 1, 3, 6, 12, and 24 months after index procedure through assessments such as the IPSS, quality of life (QoL), BPH Impact Index (BPH II), peak flow rate (Qmax), post-void residual volume (PVR), and sexual function scores. Assessed cystoscopically, PUL was found to visually increase the urethral lumen at the time of treatment. Reported adverse events were typically mild and transient and included hematuria (63 %), dysuria (58 %), and irritative symptoms (47 %). There was significant improvement in symptom scores, with a 14.2-point improvement in IPSS by 3 months and a 10.7-point improvement sustained through 1 year. This level of symptom relief is lower than that typically reported for TURP though two to three times that of medical therapy [4]. Further, LUTS-related quality of life, as measured by the QoL and BPH II, improved significantly (*P* < 0.001 throughout follow-up). Symptom scores and quality of life measures maintained improvement at 2 years, with 9.8-, 2.2-, and 4.1-point improvement in IPSS, QoL, and BPH II, respectively. As a first clinical study of a new procedure, there was variation in the device used and the technique employed. The authors explained that the procedure was significantly refined after the first 25 subjects were treated. This improvement was seen in the rate of retreatment over 2 years, which was 20 % for the entire cohort but improved to 8 % for the 39 subjects who underwent the refined procedure.

**Table 1:** Subject baseline characteristics and published study inclusion/exclusion criteria

Publication	Number	Age (SD)	Prostate volume, ml, mean (SD)	IPSS, mean (SD)	QoL, mean (SD)	BPH II, mean (SD)	Qmax, ml/s, mean (SD)	PVR, ml, mean (SD)	SHIM, mean (SD)	MSHQ-EjD, mean (SD)	MSHQ Bother, mean (SD)	Inclusion/exclusion criteria
Roehrborn et al. [14•], Roehrborn et al. [15••], McVary et al. [16•]	140	67 (8.6)	44.5 (12.4)	22.2 (5.4)	4.6 (1.1)	6.9 (2.8)	8.9 (2.2)	85.5 (69.2)	13.0 (8.4)	8.7 (3.2)	2.4 (1.7)	Inclusion: IPSS ≥ 13, Qmax ≤ 12 ml/s, prostate volume 30–80 cm^3^. Washout of 2 weeks for alpha-blockers, 3 months for 5 ARIs, and 3 days for anticoagulants before procedure Exclusion: prior surgical treatment of LUTS, median lobe obstruction, current urinary retention, PVR > 250 ml, active infection, PSA > 10 ng/ml unless biopsy negative, cystolithiasis within 3 months, bacterial prostatitis within 1 year
Cantwell et al. [17]	53	64 (8.0)	40.3 (9.9)	23.3 (5.5)	4.5 (1.2)	6.3 (3.0)	8.8 (4.2)	67.8 (66.4)	12.8 (8.3)	9.5 (10.0)	2.5 (1.7)	Inclusion: at least 50 years old, informed consent, no prior surgical BPH treatment, washed out alpha-blockers and 5 ARIs. IPSS ≥ 13, Qmax ≤ 12 ml, prostate volume between 30 and 80 cm^3^ without an obstructing median lobe Exclusion: current urinary retention, PVR > 250 ml, active infection, PSA > 10 ng/ml unless negative biopsy, cystolithiasis within 3 months, bacterial prostatitis within 1 year
Shore et al. [18•]	51	66 (7.6)	41.3 (11.6)	21.5 (5.4)	4.6 (1.0)	6.7 (3.1)	8.2 (2.2)	77.1 (74.9)	16.5 (7.3)	10.0 (2.6)	1.8 (1.4)	Inclusion: at least 50 years old, informed consent, no prior surgical BPH treatment, washed out alpha-blockers and 5 ARIs. IPSS ≥ 13, Qmax ≤ 12 ml, prostate volume between 30 and 80 cm^3^ without an obstructing median lobe Exclusion: current urinary retention, PVR > 250 ml, active infection, PSA > 10 ng/ml unless negative biopsy, cystolithiasis within 3 months, bacterial prostatitis within 1 year
Woo et al. [12], Chin et al. [13]	64	67 (7.3)	51 (23)	22.6 (5.4)	4.9 (0.9)	7.2 (2.9)	8.3 (2.2)	89 (86)	18.2 (4.9)	10.6 (2.1)	1.5 (1.4)	Inclusion: IPSS ≥ 13, Qmax 5–12 ml/s, PSA < 10 ng/ml, PVR volume <250 ml Exclusion: PSA > 10 ng/ml, history of urinary retention, previous prostate surgery, compromised renal function, current infection
McNicholas et al. [19]	102	68 (10)	48 (21)	23.2 (6.1)	4.7 (1.0)	NA	8.7 (4.0)	NA	NA	NA	NA	Inclusion: IPSS > 12, prostate volume < 60 cm^3^, Qmax < 15 ml/s, PVR < 350 ml
Abad et al. [20]	20	74.3 (NA)	42.6 (NA)	26.7 (6.0)	NA	8.4 (2.3)	8.6 (2.9)	NA	NA	NA	NA	Inclusion: at least 50 years old, IPSS > 20, Qmax < 15 ml/s, PSA < 10 ng/ml Exclusion: obstructed median lobe, urinary tract infection

*NA* not applicable

The largest trial to date of PUL therapy was the randomized, controlled, blinded L.I.F.T. study (Luminal Improvement Following Prostatic Tissue Approximation for the Treatment of LUTS secondary to BPH) of 206 subjects at 19 centers in the USA, Canada, and Australia [14•]. Subjects were at least 50 years old, had IPSS score of at least 13, and were randomized 2:1 to either PUL procedure or sham control (Table 1). In North America, the majority of subjects were treated under local anesthesia. Subjects returned to preoperative activity level by 8.6 ± 7.5 days. Adverse events included dysuria (34 %), hematuria (26 %), pain (26 %), and urgency (7 %). PUL was found to be significantly more effective at reducing symptoms than control therapy, with the PUL cohort reporting 88 % greater reduction in symptoms (*P* = 0.003). Further, PUL effects were significantly better than control with regard to peak flow rate, QoL, and BPH II. IPSS was reduced by 11.1 points (50 %) at 3 months and 10.8 points (49 %) at 1 year. Peak urinary flow rate increased 4.4 ml/s (64 %) at 3 months and was sustained to a 4.0-ml/s (59 %) improvement at 12 months. Recently released longer term follow-up of these subjects at 2 years indicates their symptom relief is largely sustained, with a 9.2-point (42 %) IPSS improvement and a 4.2-ml/s (58 %) increase in urinary flow rate at 2 years [15••]. Re-intervention for insufficient response was 5 % at 1 year, rising to a cumulative 7.5 % by 2 years.

The subjects in the control arm of the L.I.F.T. pivotal trial were allowed to crossover to active PUL treatment after unblinding occurred at 3 months. Fifty three of the 66 control subjects (80 %) met the criteria and elected to crossover (Table 1). Adverse events included dysuria (36 %), hematuria (26 %), pain (21 %), urgency (8 %), and urinary retention (8 %) [17]. The symptom relief achieved by these crossover procedures was found to be significantly greater than the self-controlled data from the same subjects after sham procedure and similar to the original cohort response (11.1- and 8.7-point improvement at 3 and 12 months, respectively). Quality of life and BPH II followed the IPSS response, as expected. Urinary flow rate improved 2.4 ml/s after sham and further improved 2.5 ml/s at 3 months after PUL. At 12 months, the cumulative improvement in flow rate of 4.6 ml/s when compared to pre-index (sham control) procedure was similar to the randomized study result. Similarly, the cumulative IPSS improvement when compared to true baseline before sham control was 10.6, again very similar to the 10.8 improvement in the randomized study. Investigators surmised that some residual effect of sham remained at point of crossover, and the sustained flow rate improvement 3 months after sham procedure may be a result of a dilatory effect from rigid cystoscopy. After PUL treatment, one patient (1/53, 2 %) required re-intervention within the first year.

In addition to highly controlled clinical trials, one publication describes experience with PUL in standard urologic practice [19]. An international retrospective study on 102 consecutive patients across 7 centers in 5 countries was conducted on subject data that was collected prospectively (Table 1). No procedure or results were omitted. Assessment timing and parameter collection varied at each center but often included IPSS, QoL, BPH II, Qmax, and PVR through a median follow-up period of 1 year. Patients received an average of 4.5 implants in prostates of volume 16 through 149 cm^3^. Adverse events included dysuria (25 %), hematuria (16 %), and urgency (10 %) with single cases of retention, urinary tract infection, and orchitis. IPSS improved 12.6 points at 3 months and 12.3 points at 12 months [19]. Qmax was statistically elevated at all time points and remained 4.0 ml/s improved from baseline at 12 months. Seven patients presented in urinary retention prior to PUL and were catheter free at the time of reporting (mean 8.3 months). A total of 4 patients (4/102, 6.5 %) progressed to TURP during the study.

A single-arm prospective study of 51 patients was conducted at 7 US centers to better elucidate the ability to conduct PUL under local anesthesia, as well as to quantify the rate of recovery and improvement in symptoms [18•]. All procedures were successfully completed under local anesthetic protocols. Pain visual analog scores indicated that discomfort with PUL was similar to that of rigid cystoscopy. The authors noted that flexible cystoscopy with topical gel lidocaine was a good indicator of tolerance of PUL under local anesthesia. Mean catheterization rate (20 %) and return to pre-operative activity (5.1 days) were improved from the L.I.F.T. study at these sites. Patients reported a mean of 2.8 days missed work to undergo PUL, and work productivity was very high at 1 month post-PUL.

**Fig. 1:**
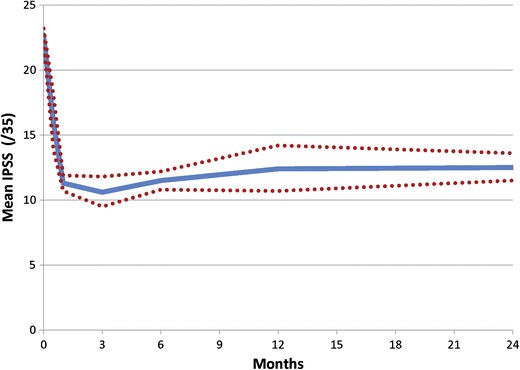
Aggregate IPSS response after treatment with PUL from six cohort studies shows rapid and durable symptom relief through 2 years. *Dotted lines* represent 95 % confidence intervals. *IPSS* International Prostate Symptom Score

A number of other studies supported the data from these initial trials [20, 21]. Individual and aggregate data demonstrate rapid symptomatic improvement that is sustained to at least 2 years (Fig. 1). Adverse events are mild to moderate and typically resolve within 2 weeks. Early PUL procedures were conducted under general anesthesia, but after further development, later studies demonstrated the ability to offer PUL as an outpatient procedure, often using only local anesthesia regimens. Average symptom relief, quality of life, BPH Impact Index, return to pre-operative activity, peak flow rate improvements, and re-intervention rates were found to be comparable among published studies (Table 2).

**Table 2:** Effectiveness of PUL as demonstrated through International Prostate Symptom Score (IPSS), quality of life (QoL), BPH Impact Index (BPH II), and peak flow rate (Qmax) change from baseline through 2 years from published studies

	2 weeks	1–1.5 months	3 months	6 months	1 year	2 years
IPSS						
# studies	3 [14•, 15••, 17, 18•]	3 [14•, 15••, 17, 18•]	2 [14•, 15••, 17]	2 [14•, 15••, 17]	2 [14•, 15••, 17]	1 [14•, 15••]
Total patients	244	244	193	193	193	140
Change from baseline	−4.5	−10.2	−11.1	−10.8	−9.8	−9.2
95 % CI	−5.5, −3.5	−11.1, −9.4	−12.2, −10.1	−11.9, −9.7	−11.7, −8.0	−10.7, −7.8
*P* value	<0.0001	<0.0001	<0.0001	<0.0001	<0.0001	<0.0001
% improvement	−18	−45	−49	−47	−45	−42
QoL						
# studies	3 [14•, 15••, 17, 18•]	3 [14•, 15••, 17, 18•]	2 [14•, 15••, 17]	2 [14•, 15••, 17]	2 [14•, 15••, 17]	1 [14•, 15••]
Total patients	244	244	193	193	193	140
Change from baseline	−1.2	−2.1	−2.2	−2.3	−2.2	−2.2
95 % CI	−1.5, −0.80	−2.3, −1.8	−2.5, −2.0	−2.6, −2.1	−2.5, −2.0	−2.6, −1.9
*P* value	<0.0001	<0.0001	<0.0001	<0.0001	<0.0001	<0.0001
% improvement	−22	−43	−47	−49	−49	−48
BPH II						
# studies	3 [14•, 15••, 17, 18•]	3 [14•, 15••, 17, 18•]	2 [14•, 15••, 17]	2 [14•, 15••, 17]	2 [14•, 15••, 17]	1 [14•, 15••]
Total patients	244	244	193	193	193	140
Change from baseline	−0.1	−3.0	−3.8	−4.0	−3.6	−3.8
95 % CI	−0.7, 0.5	−3.4, −2.6	−4.2, −3.3	−4.4, −3.5	−4.4, −2.7	−4.4, −3.1
*P* value	0.85	<0.0001	<0.0001	<0.0001	<0.0001	<0.0001
% improvement	21	−38	−55	−58	−55	−56
Qmax						
# studies		1 [18•]	2 [14•, 15••, 17]		2 [14•, 15••, 17]	1 [14•, 15••]
Total patients		51	193		193	140
Change from baseline		3.3	3.8		3.3	4.15
95 % CI		2.1, 4.5	3.1, 4.5		1.9, 4.8	3.1, 5.2
*P* value		<0.0001	<0.0001		<0.0001	<0.0001
% improvement		47	50		49	58
PVR						
# studies	2 [12, 13, 19]	2 [12, 13, 19]	4 [12, 13, 14•, 15••, 17, 19]	2 [12, 13, 19]	4 [12, 13, 14•, 15••, 17, 19]	1 [12, 13]
Total patients	166	166	359	166	359	64
% change from baseline	−5.7	7.7	−9.4	−19.4	0.9	63
95 % CI	−25.1, 13.7	−16.0, 31.2	−25.2, 6.4	−37.5, −1.3	−16.9, 18.7	−3, 130
*P* value	0.56	0.53	0.24	0.036	0.92	0.061

## Preservation of Sexual Function

PUL appears not to compromise erectile function as no adverse event reports of new-onset, sustained erectile dysfunction have been attributed to the PUL procedure. Further, data from the feasibility study conducted on 64 patients in Australia indicates that the erectile function rate as measured by Sexual Health Inventory for Men (SHIM) score improved after PUL treatment 2.2 points at 3 months and 1.7 points at 12 months (*P* < 0.05) [22]. Similar results were reported in a pivotal randomized study, with a 1.3-point improvement at 3 months and a 0.04-point improvement at 12 months (*P* < 0.05) [16•]. In addition, the PUL pivotal study demonstrated that those with intact erectile function at baseline demonstrated stable SHIM scores, which stands in contrast to the degradation in these subjects reported after laser vaporization [23, 24]. For PUL, a linear regression of change vs. baseline condition showed improvement in SHIM as baseline conditions worsen so that for men entering with severe erectile dysfunction, SHIM was significantly improved at 12 months (*P* < 0.05). The data from the PUL studies is pointedly different from the TURP results, where new-onset erectile problems have been reported in 12 % of patients [25]. This rate has been shown to be higher than the expected degradation associated with aging during study follow-up [26].

In addition to potency, ejaculatory function has been found to significantly influence a patient’s sex life [27]. In a recent review, Sturch et al. purport that many men would accept a reduction in treatment efficacy to preserve ejaculation [28]. An aspect of ejaculation that is often compromised by surgical BPH treatment is the emission of ejaculate during sexual activity. Unlike TURP, treatment with the PUL system showed no compromise in regard to the ability to emit ejaculate. There have been no adverse event reports of new-onset, sustained dry emission (anejaculation or retrograde ejaculation) attributed to the PUL procedure. Further, in the feasibility study, ejaculatory function as measured by the Male Sexual Health Questionnaire for Ejaculatory Dysfunction (MSHQ-EjD) function score increased slightly at all time points and was statistically significant at 6 weeks (1.7 points) and 3 months (1.6 points) [22]. In the larger pivotal study, ejaculatory function was similarly statistically improved at each time point during follow-up, with a 2.2-point increase at 3 months and a 1.3-point increase at 12 months. Individual MSHQ-EjD element analysis also revealed that frequency, strength, and volume of semen were also statistically improved at each time point [16•].

## Conclusion

The prostatic urethral lift is a new option for the treatment of LUTS secondary to BPH. It offers rapid relief of LUTS with a lower risk of morbidity than standard interventional options. Multiple studies have demonstrated symptom relief that may initiate within 2 weeks and be potentially sustained through 2 years. LUTS-related quality of life, as measured by the IPSS quality of life question and the BPH Impact Index, also improves after PUL. Randomized and open-label studies have demonstrated that PUL can be delivered using local anesthesia (intraurethral and oral medications) with acceptable patient comfort. Post-operative catheterization, when tested via void trial, has been shown to be 20 to 30 % with an average duration of less than 1 day.

Durability of the PUL system treatment has been demonstrated to 2 years with a mean IPSS improvement of 47–49 % at 1 year and 42–45 % at 2 years, indicating a stable response [13, 15••]. Re-intervention rates for disease progression have to date remained low, occurring in approximately 7.5 % of patients at 2 years [15••].

Sexual function appears to be preserved after PUL with no reported incidence of new-onset, sustained erectile dysfunction or dry emission with ejaculation. PUL may even improve sexual performance in patients, as average SHIM and MSHQ-EjD function scores increased after treatment in multiple studies. Ejaculatory function can be compromised after pharmacologic and interventional LUTS treatments, an issue which has received overdue attention and represents an important quality of life aspect for patients. Because no tissue is destroyed or removed with PUL and ejaculatory function is preserved, it may be uniquely positioned to address BPH LUTS while simultaneously preserving sexual function.

Although recently approved in the USA for BPH LUTS treatment, this therapy (PUL) has been under investigation since 2005 and has been reported in numerous clinical studies. The breadth, depth, and repeatability of PUL data through multiple international trials with varying investigator experience, patient populations, and health care systems indicate that the outcomes after PUL are not setting dependent and support the technology in the treatment of real-world patient settings.

Because PUL is well tolerated and associated with few risks in comparison to most interventional LUTS alternatives, it may appeal to men who are earlier in their BPH disease progression. Moreover, instead of remaining on palliative medications that may have bothersome side effects, these men may elect a minimal risk solution that is potentially more effective and durable and has the potential to preserve their sexual function. The ability to beneficially affect a population of younger men who traditionally would have received no therapy or marginally tolerated pharmacological therapy may represent a paradigm shift for interventional BPH therapy with the advent of PUL.
